# A Systematic Review and Meta-Synthesis of Barriers and Facilitators of Help-Seeking Behaviors in South Asian Women Living in High-Income Countries who Have Experienced Domestic Violence: Perception of Domestic Violence Survivors and Service Providers

**DOI:** 10.1177/15248380221126189

**Published:** 2022-10-15

**Authors:** Razia Sultana, Busra Ozen-Dursun, Omolade Femi-Ajao, Nusrat Husain, Filippo Varese, Peter Taylor

**Affiliations:** 1 University of Manchester, Oxford Road, UK

**Keywords:** domestic violence, battered women, disclosure of domestic violence, cultural contexts, perceptions of domestic violence, South Asian, help-seeking behaviours

## Abstract

There has been little research on domestic violence (DV) within ethnic minority communities in high-income countries. This study reports on the findings of a meta-ethnography that examined the barriers and facilitators of help-seeking behaviors in South Asian women living in high-income countries who have experienced DV to inform practice, understand the limits of the evidence, and identify research gaps. Qualitative studies were identified which were available in English by electronic databases. After an initial search, 2,465 articles were reviewed by title and abstract and 135 articles were reviewed for full text. Thirty-five papers were included for this review and were synthesized using meta-ethnography. Key findings included barriers and facilitators of help-seeking behaviors: (1) Socio-cultural norms to prohibit help-seeking behaviors, (2) Fear of negative consequences, (3) Negative aspects of immigration status, (4) Insufficient support from statutory, and voluntary agencies, (5) Safety strategies and facilitators for surviving. Although this review investigated the perceptions of two different populations (survivors and service providers) both groups had similar views about the barriers and facilitators of help-seeking behaviors. It is crucial for the government and non-government organizations to understand the barriers for women who are DV survivors to seek help from their organizations and also from South Asian ethnicities. The awareness and understanding of these barriers and facilitators may help support the development of interventions to encourage effective help-seeking amongst South Asian women affected by DV. Suggestions for research, practice, and policies are discussed.

## Introduction

Domestic violence (DV) is a major public health problem that affects one-third of women in the world (García-Moreno et al., 2013), and is also a silent problem among South Asians in high-income countries (Soglin et al., 2020). The term South Asian refers to people from the Indian subcontinent, for example, India, Pakistan, Bangladesh, Nepal, Bhutan, Sri Lanka, Afghanistan, and Maldives (SAARC, 2020). South Asians are the largest ethnic minority group in the United Kingdom with a population of 4 million (Office for National Statistics, 2011). South Asians are one of the most rapidly growing ethnic minority groups in the United States with over 5.4 million people (Strengthening South Asian Communities in America, 2019). Despite being a large ethnic minority community in high-income countries like the United States and United Kingdom, little attention has been paid to research on DV among South Asians.

Statistics on the prevalence of DV in South Asian women are reported as being between 21% to 40% of South Asian women living in the United States (Soglin et al., 2020). Besides, approximately 3.4% of South Asian women who were born in any of the selected South Asian countries residing in the United Kingdom experienced DV (Office for National Statistics, 2018). Evidence suggests that DV incidents against South Asian women in high-income countries are often unreported and unrecorded (Midlarsky et al., 2006). South Asians represent an intersection of diverse identities and challenges (religion, socio-cultural norms, race, ethnicity, language, migration), which can affect help-seeking amongst those who experience DV (Al’Uqdah et al., 2016; Bent-Goodley, 2007; Burnette, 2015). There is very little intersectional research conducted on the reasons for not reporting abuse amongst South Asian women (Bhuyan et al., 2010; Kapur et al., 2017). In addition, there remains a dearth of research regarding the service providers’ and survivors’ views on barriers and facilitators of help-seeking for DV (Eastman et al., 2007). It has been suggested that white women in high-income countries were approximately twice as likely to seek help for DV compared to ethnic minority women in high-income countries (Kaukinen, 2004). South Asian women were often reluctant to disclose DV matters to the police or service provider organizations for traumatic experiences with the statutory service compared to other ethnic women (Gill, 2004; Imam, 1994; Patel, 2003).

The Barriers Model (Grigsby & Hartman, 1997) was developed to understand women’s DV experiences at four different levels: barriers in the environment, family and the social role expectations, psychological consequences of abuse, and witnessing DV as a child or having been revictimized. Processes and experiences at each level may create constraints to seeking help. This model helped to explore the cluster of barriers for survivors to disclose abuse and seek help. Previous research applied this model as an integrated strategy for psychological intervention with survivors of DV (Apatinga & Tenkorang, 2021; Grigsby & Hartman, 1997). In this review we adopt ‘The Barriers Model’ to help understand the barriers and facilitators of help-seeking behaviors in South Asian women living in high-income countries who have experienced DV.

While past narrative reviews around DV in South Asian women exist, this systematic review is the first to investigate the barriers and facilitators of help-seeking behaviors, specifically in South Asian women living in high-income or developed countries who have experienced DV. Most of the literature about DV against South Asian ethnic minority women has used qualitative methods (Finfgeld-Connett & Johnson, 2013). Qualitative methods can provide a means of developing a rich and detailed understanding of this topic that is sensitive to the broader cultural and social context of these women. Therefore, this review will focus on qualitative research as this review extracted and analyzed the data from qualitative research based on DV survivors’ lived experiences and the service providers’ perception because triangulation between these multiple sources of information is beneficial in better understanding the barriers and facilitators to help-seeking for DV that South Asian women face. This systematic review aims to understand the barriers and facilitators of help-seeking behaviors in South Asian women who experience DV in high-income countries. The review focuses on studies that include the perception of DV survivors and service providers who work with them in order to accumulate insights from each of the two groups, which, taken together, would provide a more complete scenario of barriers and facilitators of help-seeking behaviors for DV than would analysis with a single perspective.

### Method

A systematic review of qualitative studies and a meta-ethnography (Noblit et al., 1988) was conducted to examine literature for understanding the barriers and facilitators of help-seeking behaviors in South Asian women who experience DV in high-income countries. This review was followed by the Preferred Reporting Items for Systematic Reviews and Meta-Analyses (PRISMA) guidelines (Page et al., 2021). This review was pre-registered in PROSPERO databases (CRD42020161228).

### Search Strategy

A systematic search was conducted on the CINAHL (Cumulative Index to Nursing and Allied Health Literature), PsycINFO, Applied Social Sciences Index and Abstracts (ASSIA) databases up to 01 May, 2022. There was no restriction on the publication date. The systematic search strategy had two main concepts: DV and South Asian women. Search Terms were as follows: (“Domestic Violence” OR “family Violence” OR “intimate partner violence” OR “Battered Women” OR “Battered Females” OR “IPV”) AND (“South Asia*” OR “Ethnic* Identity” OR “Minority* Groups” OR “Bangl*” OR “Bengal*” OR “Pakistan*” OR “India*” OR “Bhutan*” OR “Sri Lanka*”, “Afghanistan*” OR “Maldives*” OR “Nepal*”). In addition, the reference lists from included articles were hand-searched for further potentially eligible studies.

### Eligibility Criteria and Study Selection

The National Institute for Health and Care Excellence (NICE) definition of DV refers to “Any incident or pattern of incidents of controlling behavior, coercive behavior or threatening behavior, violence or abuse between those aged 16 or over who are family members or who are or have been, intimate partners. This includes psychological, physical, sexual, financial and emotional abuse. It also includes “honor’-based violence and forced marriage” (NICE, 2016, p. 5). This definition includes issues specific to ethnic communities, for example, honor-based violence and forced marriage. This definition was selected because it covers the multifaceted characteristics of DV against women.

The inclusion criteria for papers considering views of DV survivors are given: (1) Participants are all women (or identify as women). Studies with mixed-gender samples must have provided results separately for women; (2) Participants must be living in the high- income countries as defined by the World Bank (High Income Countries Population, 2019); (3) The sample must consist of women with a South Asian background (any generation will be included). If the sample in a study includes a mixture of ethnicities, then data pertaining to South Asian women data should be presented separately from the other data; (4) In this review, participants’ age will be 18 years or above because the target group in this review is adult women who have experienced DV; (5) Articles need to provide data relating to the process or experience of help-seeking.

The inclusion criteria for papers considering views of service providers are as follows: (1) Participants must be living in high-income countries as defined by the World Bank (High Income Countries Population, 2019); (2) Service providers who had the experience to provide support to adult South Asian women (18 years or above) who experienced DV; (3) Articles need to provide data relating to the process or experience of help-seeking.

**Figure 1.**
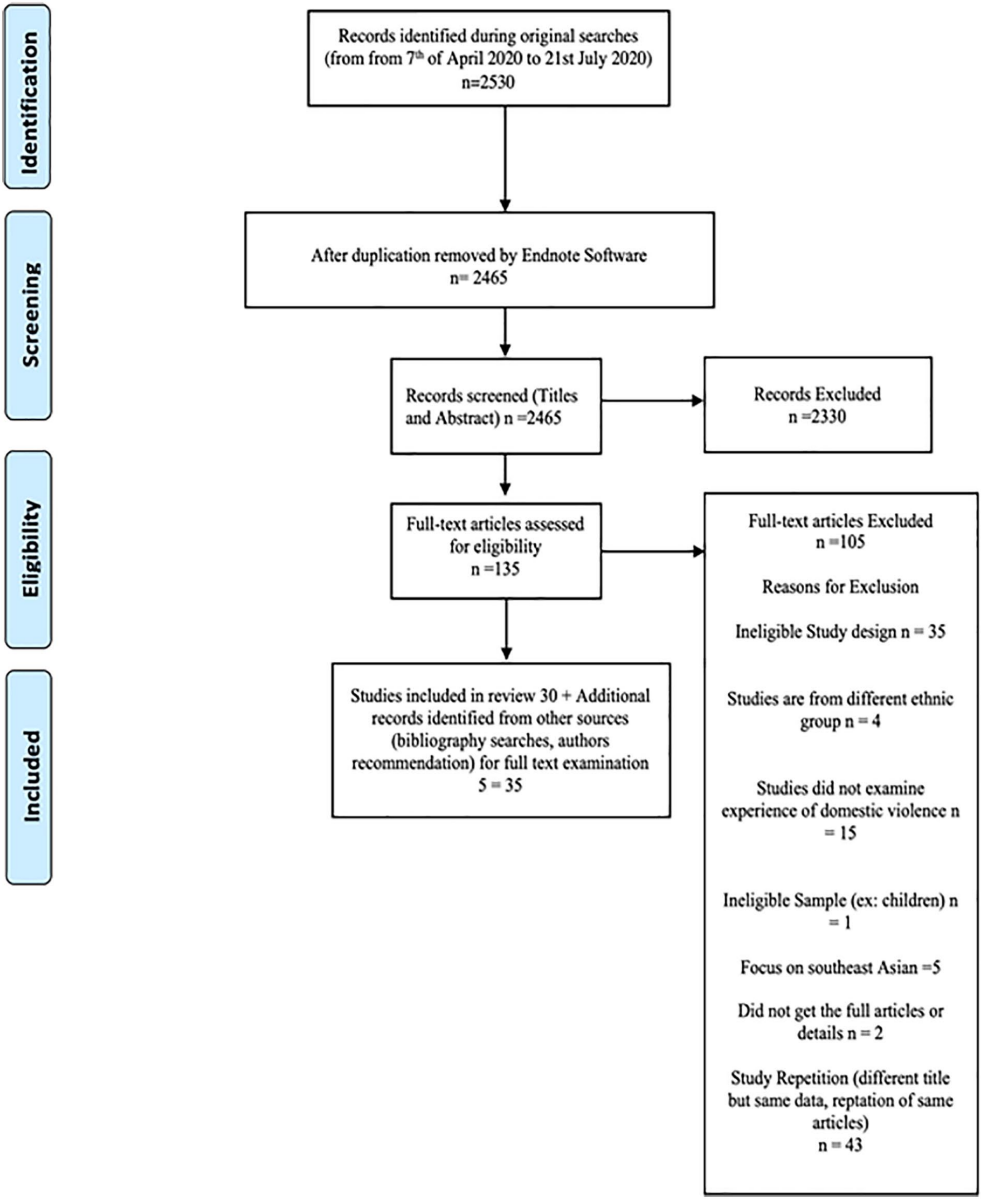
Literature search PRISMA diagram. *Note*. PRISMA = Preferred Reporting Items for Systematic Reviews and Meta-Analyses.

English language studies must include with a purely qualitative design or a mixed method design that includes a qualitative component. Title/Abstract screening and full-text screening were conducted by two reviewers independently. Any disagreement between the two reviewers was resolved by discussion. Also, the final author consulted if the reviewers did not reach an agreement. The study selection is presented in the flow chart (See Figure 1).

### Data Extraction

The reviewers extracted data in parallel from each study into a pre-developed data extraction form. First author name, publication year, country, sample size, participants’ age, and methods of data collection and analysis were extracted from the studies and summarized in Table 1. Additionally, more information about the study characteristics could be seen in Table 2.

**Table 1.** Critical Findings (Third Order Constructs, Summary of Second Order Constructs and Examples of First Order Constructs).

Third Order Construct	Summary of Second Order Construct	Examples of Quotes (First Order Construct)
Socio-cultural norms to prohibit help-seeking behaviors: Data extracted from 30 studies: 16(S), 7(SP), 7(SSP)	• Normalizing abuse • Family secret • Stigma around seeking help • Gender inequality	“Even if you get to complain or get help from others, it won’t be changed, because that is his nature and a husband who is drinking everyday cannot change his behavior. Seeking help creates problems due to misunderstanding. Accept the drinking time if at other times he is ok” (Kanagaratnam et al., 2012, p. 651)
		“It is not socially accepted that women get divorce. It is the reason why women will not initiate divorce. The situation is, whether the woman applies for divorce or whether the husband leaves the wife, the woman is the one to be blamed for the breakup of the marriage . . . yeah; women get the blame if there is problem in the marriage . . . and shame it brings . . .” (Tonsing & Barn, 2017, p. 633)
Fear about family, community, friends data extracted from 28 studies: 15(S), 7(SP), 6(SSP)	• Deportation Threat/risk • Silent Approval of the violence for immigration issues • Lack of knowledge, resources and cultural barriers for immigrant women	“I know if, (deep breath and short pause) I decide to leave (husband), he’s gonna go off on the deep end, he’s gonna do something bad. . .He’ll kill me, like I don’t know. I mean in our, in our Indian culture that’s what happens sometimes” (Hunjan, 2004, p. 164) “I do not share with others because if I share with someone, then that someone might tell another person who might happen to know my mother-in-law and so on. And the news will spread and it will bring bad name to my family . . .” (Tonsing & Barn, 2017, p. 632) “continuously this person was like chasing me. . . So what, why I took this decision to come another country, because, although my family was supportive, in the end, I became a liability for them. . .” (Hunjan, 2004, p. 187) The following image is that of a dependent victim who is “not able to work, unable to drive. . . doesn’t really get out of the home and then there are all these things that are happening, they might have children together, he is threatening to send her back to India. He will keep the kids here with him, [and] she is unfamiliar with her options for UK visa” (Kapur & Zajicek, 2018, p. 1935)
Negative aspects on immigration status: Data extracted from 24 studies: 13(S), 6(SP), 5(SSP)	• Being financially controlled • No public fund	“There is fear of income insecurity, how to pay for rent and food for kids.” Another participant added “Where to leave kids when you go to work” (Ahmad et al., 2009, p. 617) “I didn’t know what are my rights. . . . Migrants don’t have any information . . . the Swedish people they can just bring the person from the other place. Because they don’t know the language they can give the information or not. They can build the whole situation for that person. It is a new form of slavery. . . . You have not language, you have not contacts.” (Voolma, 2018, p. 1843) “Some of the interpreters that are available may not be sympathetic to the issues and may try to twist her word around”(Huisman, 1996, p. 268)
Insufficient support from statutory and voluntary agencies: Data extracted from 19 studies: 10(S), 5(SP), 4(SSP)	• Lack of coordinated services • Institutional racism	“There is a lot of resistance among our clients . . . we did a survey with 50 or more of our clients, asked them their feelings about shelter, police, and other things . . . with shelter a very small percentage would ever be willing to call or go to a shelter and part of that is because mainstream doesn’t understand me. I cannot get Halal food or whatever food . . . So there is a huge . . . resistance among our clients in accessing services/spaces.” (Kapur et al., 2017, p. 56)
Safety strategies and facilitators for surviving: Data extracted from 22 studies: 13(S), 4(SP), 4(SSP)	• Support by families, friends, community and society • Self-disclosure and self help • Statutory and voluntary agencies support from the host countries	“Through speaking with the counsellors, I have learnt a lot and heard a lot. I can now do a lot. Formerly, in the first year and half, I could not go out of the door alone. In the past six months I have learnt a lot. I can now travel alone and I am not scared anymore. . .” (Hunjan, 2004, p. 193) “The police, as authority figures, are seen next to God, because they have the power .. . But if the police have no understanding of the issue, their advice would not be very useful. There should be more recruitment of BME officers who will understand these cultural complexities, and there should be more training in cultural and sensitive issues.” (Belur, 2008, p. 437)

*Note*. S = survivor based studies; SP = service provider based studies; SSP = survivor and service provider based studies.

**Table 2.** Characteristics of Included Studies.

Author Name and Year	Country	Sample Size	Age Range	Method of Data Collection	Method of Data Analysis and Theories Used
Ahmad et al. (2009)	Canada	22	29 to 68 (*M* = 45.9)	Focus group	Thematic analysis
Ahmad et al. (2013)	Canada	11	32 to 57	Interview	Thematic analysis
Ali et al. (2019)	Pakistan and England	41	20 to 62	Interview	Grounded theory methodology
Anitha (2010)	Manchester, UK	30	Age is not mentioned	Interview	Thematic analysis
Belur (2008)	England and Wales	Clearly not mentioned: Only mentioned two police forces	Age is not mentioned	1. Interview 2. Observation	Observational research technique
Bhandari (2020)	United States	40	26 to 66 (*M* = 38.8)	Interview	Open coding strategy defined by Saldana (2012)
Bhandari and Sabri (2020)	United States	20	26 to 66 (*M* = 38.8)	Interview	Open-coding strategy as defined by Saldana (2012)
Bhuyan (2008)	Washington, United States	No information	Age is not mentioned	Ethnographic methods, observation, interviews	Discourse analysis
Dasgupta and Warrier (1996)	United States	12	Age is not mentioned	Interview	Not mentioned clearly, assumed narrative
Garnweidner-Holme et al. (2017)	Norway	3	Age is not mentioned	Interview	Thematic analysis
Guruge and Humphreys (2009)	Canada	16	30+	Interview and focus groups discussion	Inductive thematic analysis
Guruge et al. (2010)	Canada	63	Community leaders age group (Individual Interview): 6 (in their 30s), 5 (in their 40s), 5 (over 50 years, Focus Group discussion = G1: 30 to 63, G2: 41 to 50 G3: 27 to 65, G4: 25 to 62, G5: 24 to 69, G6: 35 to 69	Interview and focus group discussions.	Data analysis involved inductive thematic analysis (Bryman, 2016)
Hunjan (2004)	Canada	Clearly not mentioned	20 to 53 (*M* = 33.7)	Interview	Not specifically mentioned, used NVivo
Kanagaratnam et al. (2012)	Canada	63	Total eight focus groups two with each of the following groups): (a) young women aged 18 to 24 who were born in Canada or immigrated at or under the age of 13, (b) adult women aged 25 to 64 who were married, (c) women older than 65 who were currently/formerly married	Focus groups	Thematic analysis
Kapur and Zajicek (2018)	United States	26	20 to 70+	Interview	Winker and Degele’s (2011) methodology of intersectional analysis
Kapur et al. (2017)	United states	26	Age is not mentioned	Interview	Grounded theory
Mahapatra and Rai (2019)	United states	9	24 to 42	Interview	The types of codes used were: Descriptive, Process, In Vivo, and Emotion coding.
Mehrotra (1999)	United States	28	27 to 54 (*M* = 40)	Interview	Narrative analysis
Mirza (2016)	UK (Scotland)	11	No information	Interview	Not clearly mentioned/Coding
Pallatino (2018)	United States	30	Age range is 20 to 35	Interviews	Not clearly mentioned/Coding
Reddy (2019)	United States	8	Age range is 26 to 49 (*M* = 32)	Interview	Phenomenological analysis
Sabri et al. (2014)	United States	16	Age range is 31 to 48 (*M* = 38)	Interview and focus group	Thematic analysis
Shah (2015)	United States	8	Age range is 30 to 50	Interview	Conventional content analysis
Shamoon (2018)	United States	8	Age range is 20 to 50	Interview	Constructivist grounded theory methods
Shirwadkar (2004)	Canada	8	No information	Interview	Not clearly mentioned/Coding
Tonsing and Barn (2017)	Hongkong	14	Age range is 27 to 39 (*M* = 33.9)	Interview	The grounded theory
Tonsing (2016)	Hongkong	20	1. Age range is 27 to 39 (*M* = 33.9) in South Asian women. 2. Age range is 20 to 35 in helping professionals	Interview	The grounded theory
Wellock (2010)	UK	6	No information	Interview	Phenomenological analysis
Gill (2004)	UK	18	No information	Interview	Not clearly mentioned/Coding
Burman et al. (2004)	UK	36	Adults	Interview	Discourse analysis
Hyman and Robin (2006)	Toronto, Canada	51	Age range is 19 to 77	Focus group	Not clearly mentioned/Coding
Huisman (1996)	United States	18	No information	Interview	Feminist perspective
Ahmadzai et al. (2016)	Canada	5	No information	Interview	Gender-based analysis plus (GBA+) framework
Voolma (2018)	Sweden, UK	88	22 to 48	Interview	Thematic analysis
Ahmad-Stout et al. (2021)	United States	11	24 to 49	Interview	The grounded theory

### Risk of Bias (Quality) Assessment

For this review, a quality assessment of included studies has been done using the Critical Appraisals Skills Programme (CASP) (MILTON, 2002) tool as this tool has been applied in several qualitative systematic reviews and found worthwhile. This CASP tool for qualitative studies enables an assessment of each article’s credibility, rigour, and relevance (Kitto et al., 2008; Kuper, Lingard & Levinson, 2008; Kuper, Reeves & Levinson, 2008). This tool covers ten domains, including the adequacy of research findings, rigour in data collection, data analysis and interpretation, ethical clarity, researchers’ engagement in reflexivity, and the value of the papers. The more “yes” responses indicate better methodological quality. The other potential responses are “can’t tell,” and “no.” Two reviewers (BO, RS) rated all papers, as per the CASP’s guidelines. Each discrepancy was discussed and resolved by the reviewers. If no consensus arose, the supervisory team discussed the discrepancy until a consensus was achieved. There was no study excluded in this stage based on quality but a discussion on the quality of the articles was included in the review, and this information was used to weigh the conclusions drawn from the data synthesis.

### Meta-Synthesis

In this review, we have used meta-ethnography as a method for synthesizing qualitative research. This method gives an alternative to traditional aggregative methods of synthesis (Britten et al, 2002). Meta-ethnography was used for this review as it introduces an integrated approach to synthesis which helps to interpret the findings and also develop conceptual understanding or theory (Britten et al., 2002; Toye et al., 2014). The synthesis process involved a series of steps (Noblit et al., 1988). First, reviewers read the articles multiple times for understanding, familiarization of the key concepts, and metaphors which were published in the studies. Next, the reviewer coded the participants’ views/perceptions about barriers and facilitators of help-seeking behaviors for DV. NVivo 12 (Version 12.6.1) software was used to assist in coding the data. All papers were uploaded to NVivo, which allows codes to be readily applied and tracked between papers. The participants’ (service providers and survivors) quotes and authors’ interpretations were coded line by line as a single body of sentences for creating a distinction between first-order, second-order, and third-order constructs (Tarzia et al., 2020). The views and experiences of study participants (survivors and service providers) as presented in the individual papers are referred to as first-order constructs. The authors’ explanations and interpretations of the data obtained within their studies are referred to as second-order constructs. The overarching themes identified through the meta-synthesis from across the different primary studies are referred to as third-order constructs (Noblit et al., 1988; Trevillion et al., 2014).

The first author who led the coding regularly discussed the process with the second reviewer and the supervisory team to provide additional oversight and opportunities for reflection. All the codes were separated in the same table into three groups of papers: (1) Survivors-based papers, (2) Service providers-based papers, (3) Survivor and service providers (SSP)-based papers. The codes were categorized by the areas of similarity, and then all categories were merged into relevant themes and subthemes. The translation process involves conceptualizing the relationship between studies and the first and second-order constructs, which is a mandatory step in the meta-ethnography (Britten et al., 2002). In this review we used Noblit et al. (1988)’s reciprocal translation and a line of argument. The data was largely consistent (in agreement) between papers, so a refutational translation was not applicable. A summary of critical findings (second-order constructs and examples of first-order constructs) are displayed in Table 1:

## Results

### Study Selection

A total of 2465 studies were identified after removing the duplications. After the title and abstract screening, 69 studies were identified for the full-text screening. Thirty-five papers fully met the inclusion criteria and were included in this review. The total identification and screening process is shown in Figure1.

### Characteristics of Included Studies

Table 2 presents the total characteristics of the included studies.There were twenty survivor-based papers, eight service providers, and seven survivors and services. This review included 30 journal articles and five dissertations/theses. Included studies were conducted in eight different countries. Fifteen studies were conducted in the United States, eight were conducted in the United Kingdom, nine were conducted in Canada, two were conducted in Hong Kong, one was conducted in Sweden, and one was conducted in Norway. One was conducted jointly in India and United States and one was conducted jointly in Pakistan and UK. In these two studies, we have excluded the information concerning individuals living in India and Pakistan as this review focused on South Asian ethnic communities in high-income countries. Participants’ ethnicities were Indian, Pakistani, Bangladeshis, Afghan, and Sri Lankan.

The majority of studies used semi-structured interviews (24 studies) whilst eleven studies are based on focus group discussion, unstructured interviews, and observation methods. Thematic analysis was the most common method of analysis (*n* = 9). Other methods were grounded theory (*n* = 4), conventional content analysis (*n* = 1), narrative analysis (*n* = 2), and discourse analysis (*n*= 1).

Survivors were recruited from a variety of settings such as community organizations, researcher’s networks, refugee camps, mosques, and via survivors’ friends and relatives using snowballing techniques. The service providers were recruited from third-party organizations. Service providers included DV advocates, immigrants’ lawyers, community leaders, NGO workers, volunteers, founders of South Asian community organizations, and activists in non-profit organizations.

### Results of Quality Assessment

The quality assessment Table 3 is given below. The quality assessment indicated that two papers did not comment on reflexivity (i.e., the positioning and background of the research team in relation to the topic being studied), and nineteen papers had no clear information about this reflexivity which were rated as “can’t tell” in CASP tool. Reflexivity is crucial because without it, there is greater risk of personal biases not being recognized or acknowledged and impacting on the quality of the analysis (Probst, 2015). Five papers did not mention any ethical issues and eleven papers provided insufficient information about ethical issues (Sanjari et al., 2014). Data collection-related limitations were found in two papers that did not mention the sample age group, sample size, race, or ethnicity, or clarify the method of translation or data collection. Although some papers had few issues, all the papers were rated as valuable by first and second reviewers for their contribution to creating existing knowledge or understanding and identifying new areas where research is necessary.

**Table 3.** Results of Quality Assessment.

Author Name and Year	1. Was there a clear statement of the aims of the research?	2. Is a qualitative methodology appropriate?	3. Was the research design appropriate to address the aims of the research?	4. Was the recruitment strategy appropriate to the aims of the research?	5. Was the data collected in a way that addressed the research issue?	6. Has the relationship between researcher and participants been adequately considered?	7. Have ethical issues been taken into consideration?	8. Was the data analysis sufficiently rigorous?	9. Is there a clear statement of findings?
Ahmad et al. (2009)	Yes	Yes	Yes	Yes	Yes	Yes	Yes	Yes	Yes
Ahmad et al. (2013)	Yes	Yes	Yes	Yes	Yes	Yes	Yes	Yes	Yes
Ali et al. (2019)	Yes	Yes	Yes	Yes	Yes	Yes	Yes	Yes	Yes
Anitha (2010)	Yes	Yes	Yes	Yes	Yes	Can’t tell	Can’t tell	Yes	Yes
Belur (2008)	Yes	Yes	Yes	Yes	Yes	Can’t tell	Can’t tell	Yes	Yes
Bhandari (2020)	Yes	Yes	Yes	Yes	Yes	No	Can’t tell	Yes	Yes
Bhandari and Sabri (2020)	Yes	Yes	Yes	Yes	Yes	Can’t tell	Yes	Yes	Yes
Bhuyan (2008)	Yes	Yes	Yes	Yes	Yes	Can’t tell	Can’t tell	Yes	Yes
Dasgupta (1996)	Yes	Yes	Yes	Yes	Can’t tell	Can’t tell	Can’t tell	Yes	Yes
Garnweidner-Holme et al. (2017)	Yes	Yes	Yes	Yes	Yes	Can’t tell	Yes	Yes	Yes
Guruge and Humphreys (2009)	Yes	Yes	Yes	Yes	Yes	Yes	No	Can’t tell	Yes
Guruge et al. (2010)	Yes	Yes	Yes	Yes	Yes	Yes	Yes	Yes	Yes
Hunjan (2004)	Yes	Yes	Yes	Yes	Yes	Yes	No	Can’t tell	Yes
Kanagaratnam et al. (2012)	Yes	Yes	Yes	Yes	Yes	Yes	Yes	Yes	Yes
Kapur and Zajicek (2018)	Yes	Yes	Yes	Yes	Yes	Can’t tell	No	Yes	Yes
Kapur et al. (2017)	Yes	Yes	Yes	Yes	Yes	Yes	Yes	Yes	Yes
Mahapatra and Rai (2019)	Yes	Yes	Yes	Yes	Yes	Can’t tell	Yes	Yes	Yes
Mehrotra (1999)	Yes	Yes	Yes	Yes	Yes	Yes	Can’t tell	Yes	Yes
Mirza (2016)	Yes	Yes	Yes	Yes	Yes	Can’t tell	Can’t tell	Yes	Yes
Pallatino (2018)	Yes	Yes	Can’t tell	Yes	Yes	Can’t tell	Yes	Yes	Yes
Reddy (2019)	Yes	Yes	Yes	Yes	Yes	Yes	Yes	Yes	Yes
Sabri et al. (2014)	Yes	Yes	Yes	Yes	Yes	Yes	Yes	Yes	Yes
Shah (2015)	Yes	Yes	Yes	Yes	Yes	Yes	Can’t tell	Yes	Yes
Shamoon (2018)	Yes	Yes	Yes	Yes	Yes	Yes	Yes	Yes	Yes
Shirwadkar (2004)	Yes	Yes	Yes	Yes	Yes	Can’t tell	Can’t tell	Yes	Yes
Tonsing and Barn (2017)	Yes	Yes	Yes	Yes	Yes	Can’t tell	Yes	Yes	Yes
Tonsing(2016)	Yes	Yes	Yes	Yes	Yes	Yes	Yes	Yes	Yes
Wellock (2010)	Yes	Yes	Yes	Yes	Yes	Can’t tell	Yes	Yes	Yes
Gill (2004)	Yes	Yes	Yes	Yes	Yes	Can’t tell	Can’t tell	Yes	Yes
Burman et al. (2004)	Yes	Yes	Yes	Yes	Yes	Can’t tell	Can’t tell	Yes	Yes
Hyman and Robin (2006)	Yes	Yes	Yes	Yes	Yes	Can’t tell	No	Yes	Yes
Huisman (1996)	Yes	Yes	Yes	Yes	Yes	Yes	Can’t tell	Yes	Yes
Ahmadzai et al. (2016)	Yes	Yes	Yes	Yes	Yes	Can’t tell	Can’t tell	Yes	Yes
Voolma (2018)	Yes	Yes	Yes	Yes	Yes	Can’t tell	Yes	Yes	Yes
Ahmad-Stout et al. (2021)	Yes	Yes	Yes	Yes	Yes	Can’t tell	Yes	Yes	Yes

### Reciprocal Translation

It was found from the synthesis that the articles were relatively consistent with regards to their research aims and questions, and the emerging data, and as such a reciprocal translation was possible, which showed that articles contributed the most materials to this results section (Noblit et al., 1988; Sattar et al., 2021). The synthesis results in five themes, which are described below.

#### Socio-cultural norms to prohibit help-seeking behaviors

The participants described how women learned behavior from early life, following traditional gender norms where women were expected to tolerate abuse and keep it secret from the outside world (Das Dasgupta & Warrier, 1996; Hunjan, 2004; Sabri et al., 2018; Shah, 2015; Shamoon, 2018; Tonsing & Barn, 2017). Participants indicated that within South Asian society, patriarchal cultural norms left the women feeling insecure, powerless, submissive, and lacking the competency to make their own decision. This in turn made seeking help for DV very difficult (Dasgupta & Warrier, 1996; Gill, 2004; Mahapatra, 2012; Mahapatra & Rai, 2019; Shamoon, 2018).

#### Fear about family, community, and friends

Survivors feared the possible consequences for other people in their lives if they were to seek help. For example, they described concerns that the perpetrators would take revenge on their families, including those still living in the Indian subcontinent (Anitha, 2010). Survivors reported a common fear that abusers would harm their children if they tried to leave the relationship (Hunjan, 2004). It was also found that women feared divorce as the stigma and blame connected with divorce in South Asian culture, so children’s future marriage might be affected by their parent’s separation or divorce (Hunjan, 2004; Hyman, 2006; Mahapatra & Rai, 2019; Tonsing & Barn, 2017). One survivor who had managed to secure financial support for herself and her children in Canada stated that going back to her home country would have been terrible because it meant confronting the friends, family, and community members who stigmatized her for her divorce or separation (Hunjan, 2004).

#### Negative aspects of immigration status

Participants from survivor-based studies articulated that their husbands and in-laws constantly threatened to send them back to their home country (Hunjan, 2004). Perpetrators applied this “deportation threat” as a weapon to manipulate their wives into not seeking help from others (Gill, 2004). Survivor-based, service provider-based and survivor and service provider-based papers found that immigration issues are connected to exacerbating DV. Some American South Asian women reported that they tolerated violence from their husbands and in-laws as they had dependent visas where abusive husbands had the authority to get them permanent residency status (Gill, 2004; Pallatino, 2018). Immigrant women and service providers expressed that limited or no capacity to speak English was a significant barrier for South Asian and ethnic women to getting support from consultants, midwives, and police via translator or interpreter (Anitha, 2010; Garnweidner-Holme et al., 2017; Hunjan, 2004; Kapur et al., 2017) (Belur, 2008).

#### Insufficient support from statutory and voluntary agencies

Service providers stated that health, communal, and settlement organizations had limited coordination and interconnection with each other, so survivors did not get the right support at the right time (Guruge & Humphreys, 2009; Kapur et al., 2017). Both survivors and service providers-based studies narrated that immigrant women did not disclose the abuse and seek help due to their insecure visa status in the United Kingdom, and having no right to financial support, accommodation support, and welfare benefits (Voolma, 2018).

#### Safety strategies and facilitators for surviving

Some studies suggested that leaving an abusive relationship could be supported by validation from their family, friends, or work colleagues (Sabri et al., 2018; Shah, 2015). Women used their resilience as their capacity to recover quickly from their difficulties (Mahapatra & Rai, 2019), renewed their confidence and self-esteem through their positivity (Shah, 2015), and coped by engaging in positive activities (Ahmad-Stout, 2021). Moreover, they raised their voices against life-threatening torture (Bhuyan, 2008), and applied for asylum to save themselves from their husbands’ controlling patterns of behavior (Hunjan, 2004; Shamoon, 2018). Women also tried to educate themselves to understand healthy relationships (Garnweidner-Holme et al., 2017; Shamoon, 2018). Survivor women emphasized the importance of technological support such as access to computers (and mobile phones) and the internet. This enabled access to information and education through websites and online prevention programs, and provided new opportunities for communication and seeking support (email, video call, social media) (Ahmad-Stout, 2021; Ahmad et al., 2009; Mahapatra & Rai, 2019; Pallatino, 2018).

Most of the articles also showed that insufficient support existed in statutory and voluntary agencies in the host countries. However, one service provider from the United Kingdom mentioned that Asian police officers handled Asian DV issues in more effective ways due to understanding the sensitive issues in marriages, and the customs of Asian communities better than white police officers (Belur, 2008).

### Line-of-Argument Synthesis

**Figure 2.**
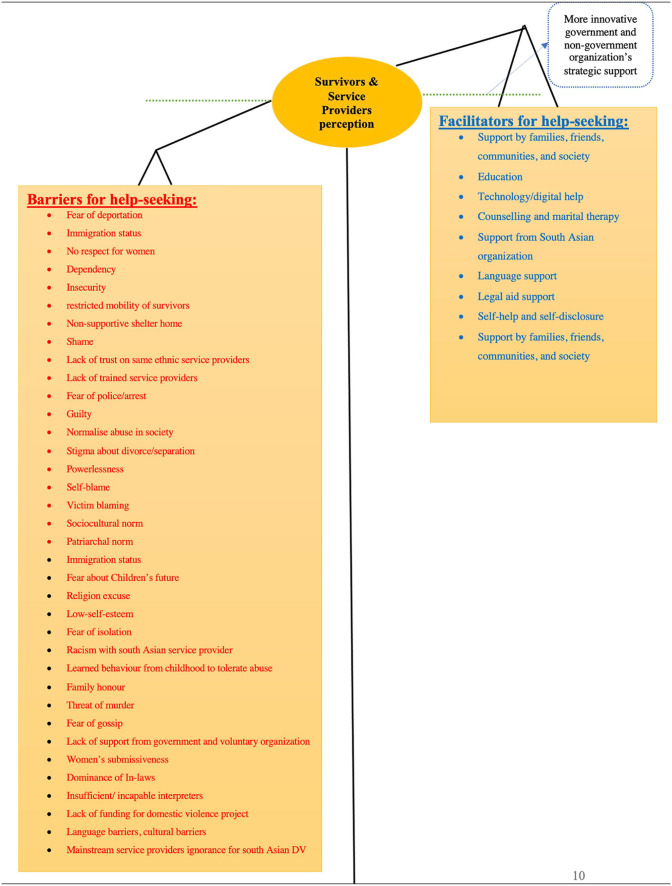
Line of argument (Scale of Weighing).

In this review, line-of-argument was created from the third order constructs which “making a whole into something more than the parts alone imply” (Noblit et al., 1988, p. 28). The findings of this meta-ethnography are illustrated in Figure 2 as a weighing scale in order to represent how the barriers and facilitators of help-seeking are balanced against each other, with the barriers outweighing the facilitators for many women struggling to seek help. The barriers to seeking help often appear to outweigh the facilitators, explaining why seeking help is so difficult for these women. It is an important issue here that South Asian women have stark cultural differences compared to the high-income countries’ cultural values and norms, creating a number of barriers with a lack of government and non-government support, which might otherwise tip the balance in favour of seeking support (Hunjan, 2004; Kapur & Zajicek, 2018; Shirwadkar, 2004). Women’s self-help and self-disclosures to protect them from the perpetrators were powerful strengths in seeking help. For some, these assets became impossible where more substantial barriers were present such as challenges concerning immigration status, financial crisis, family, and stigmatization within the community. These barriers to help-seeking for DV have occurred due to the diverse cultural attitudes of women, such as fear of shame, blame, and protection of honor of family for South Asian, and lack of formal and informal support. In Figure 2, the line-of-argument synthesis also shows a green dot line which emphasizes increasing the support for survivors and service providers to balance the ambivalent weighing scale. This balance can be ensured through more innovative, strategic assistance from government and non-government organizations.

## Discussion

**Figure 3.**
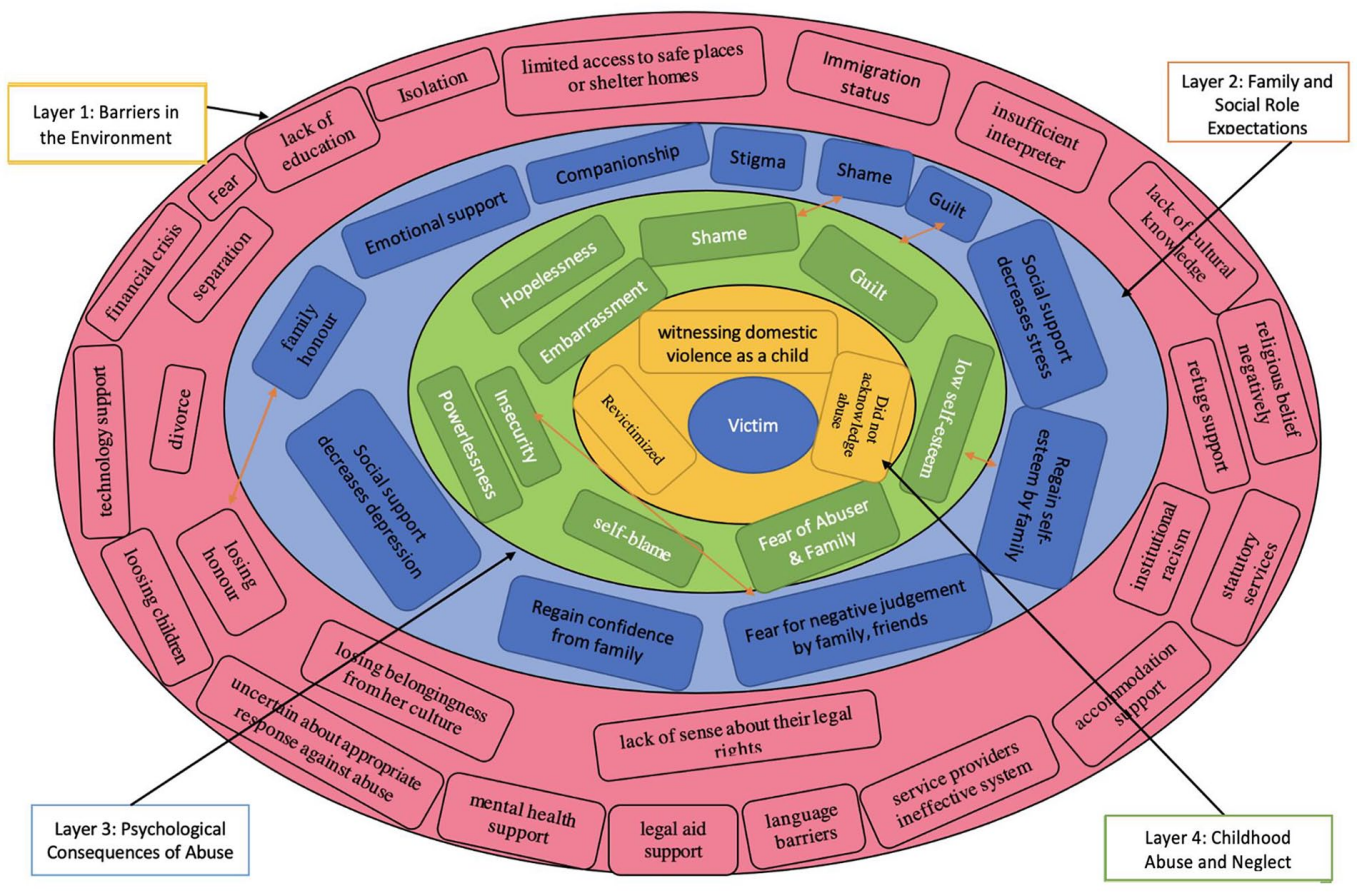
Adapted Barriers Mmodel from Grigsby and Hartman.

This meta-ethnography was the first to focus on DV survivors’ and service providers’ perceptions of the barriers and facilitators to help-seeking behaviors in the diverse group of South Asian ethnic women living in high-income countries who have experienced DV. Five themes were identified from the synthesis of qualitative research articles. Across all the themes (third-order constructs) of this review, survivors and service providers had similar perceptions about the barriers and facilitators to help-seeking behaviors for DV. The results indicated that barriers often outweigh facilitators of help-seeking behaviors. South Asian women often face a lack of support from government and non-government organizations because mainstream organizations have limitations due to their lack of funding and lack of staff trained to understand and accommodate Asian needs and experiences. The Barriers Model by Grigsby and Hartman (1997) that explains the process of help-seeking is relevant to the findings of this review. Figure 3 is adapted from Grigsby and Hartman’s (1997) Barriers Model where the help-seeking barriers from each layer overlapped/interacted with other barriers from other layers.

As mentioned earlier, this model depicts four layers at which barriers to help-seeking can exist. The themes and the barriers identified in this review can be positioned within this model. The environmental barrier is the first level, encompassing broader systemic and societal factors such as the participant’s isolation, language barriers, lack of education, lack of cultural knowledge, service providers’ ineffective systems, the financial crisis, lack of technology support, mental health support, legal aid support, discrimination regarding religious beliefs, institutional racism, insufficient interpreters, uncertainty about appropriate responses against abuse, and limited access to safe places or shelter homes. Some authors also reported this finding (Aujla, 2020; Femi-Ajao et al., 2020; Finfgeld-Connett & Johnson, 2013). South Asian women specially who were immigrants often faced these difficulties for their different culture, tradition, law, and isolation in the new countries (Abraham, 1998). The findings about the economic crises of survivor women is consistent with that of Othman et al. (2013) who reported abusers’ greater power and resources create barriers for women to seeking help as some women were financially dependent on abusers. Another important finding was that fear is a major barrier depending upon the situation (environment) which prohibits women from seeking help. Women were afraid of worsening abuse, of losing honor, divorce, separation, losing children, being deported, isolation, and being rejected from their culture (Ahmad-Stout, 2021; Gill, 2004; Hunjan, 2004). These barriers continued as the service providers could not gain access to help survivors without their permission (Ahmadzai et al., 2016; Burman et al., 2004). The negative impact of immigration status is one of the most influential environmental barriers to help-seeking and was, directly and indirectly, aligned with other barriers as previously discussed, because there were limited facilities offered for immigrant South Asian DV survivors to prevent and combat the perpetrators. These findings follow the line of other UK-based articles that showed that immigrant DV survivors who had no resources to public funds did not have access to refuge support, statutory services, accommodation support and had limited understanding of their legal rights (Alaggia et al., 2009; Femi-Ajao et al., 2020).

The second level of the model is family and social expectations. This review found that socio-cultural norms may prevent help-seeking behaviors. This level is also connected with current findings that participants were fearful of the impact on family honor if they were to seek help, as well as being worried about stigma, shame, the possibility of divorce, losing children, and the impact on children’s futures. South Asian women may also feel responsible for the violence and keep it secret from family, friends, and members of the community (St Pierre & Senn, 2010). These results seem to be consistent with other research which found survivors feared being judged negatively by their family, friends, neighbors, and colleagues (Damra et al., 2015; Hegarty & Taft, 2001; Heron et al., 2021; Lutz, 2005). In accordance with the present results, previous studies showed that the emotional support and companionship from families, friends, and communities can help some survivors regain confidence and self-esteem (Abrahams, 2007; Shah, 2015). This evidence is supported by Mahapatra (2012), who suggests that social support might minimise violence and help some survivors to minimise depression and other types of stress (Adkins & Dush, 2010; Campbell et al., 1995; Mahapatra, 2012). Psychological barriers to abuse are the third level of this barrier model, which is comparable to this review’s results, such as fear of the abuser and family; self-blame; feelings of guilt; low self-esteem; hopelessness; insecurity; powerlessness; feelings of shame; and embarrassment. These emotional struggles might make it difficult for survivors to get the courage to seek help. Relatedly, when comparing the findings from this review with Heron et al. (2021), similar barriers were found which prevented survivors from seeking help. The last level is witnessing DV as a child or having been revictimized, which are also aligned with socio-cultural norms. Some women who were victimized by someone in the family in their childhood assume that anyone can abuse them such as family members, their husband or in-laws and that they should accept it. These findings corroborate the ideas of Narula et al. (2012) and Zink et al. (2004) who suggest that some survivors did not seek help as they did not acknowledge that they were experiencing violence.

The above Barriers Model organized the themes of this review into a structure, and explained that barriers to help-seeking behaviors contributed at multiple different levels. So, the structure of the Barriers Model can be useful in understanding DV survivors’ barriers to help-seeking behaviors and may suggest strategies to address survivors' needs more effectively (Grigsby & Hartman, 1997).

### Limitations

There are a few limitations that exist in this meta-ethnography and the included studies. Although this review is about DV against South Asian women from high-income countries, only English language papers were selected for this review and this review did not include a thorough search of grey literature. As such, relevant research may have been missed, including non-English language publications. The analysis section of this meta-ethnography was undertaken by one reviewer so there might be scope for bias in the interpretations drawn. However, the research actively discussed the synthesis as it developed within regular supervision, providing an opportunity to reflect on the position taken and interpretations drawn. There is a possibility of bias given the subjective nature of qualitative research, which possibly shaped the results of this current review paper (Heron et al., 2021). Participants in any research on a sensitive topic (e.g., DV) might answer the questions based on what is accepted by society instead of what he or she originally felt. Alternatively, bias can come from researchers if they unconsciously interpret the data to meet their aims or include the data which they think is relevant for their research. The current review used the CASP (MILTON, 2002) to support the quality assessment of papers. However it has been noted that there is no gold standard quality assessment yet published for qualitative research (Mohammed et al., 2016).

The quality assessment found that some articles were affected by methodological weaknesses, ethical issues, and a lack of reflexivity. Further, it was mentioned in the articles that all South Asian cultures are not the same in terms of their religions, and social norms, and each South Asian woman may have characteristics which are distinct from other South Asian women (Ahmad et al., 2009). Studies included in this review were carried out in high-income countries, so the findings of this review will be less applicable to lower and middle-income counties.

### Implications for Policy, Practice and Research

This findings of this meta-ethnography have implications for staff of government and non-government organizations, patients, victims, and survivors of DV in understanding the barriers and facilitators to adequate support and services. Service providers must have expertise in cultural sensitivity and gender sensitivity when working with South Asian women experiencing DV in high-income countries. One well-known DV-based research project investigated gender sensitivity among social workers who provided support to immigrant DV survivors and found that failure to practice gender sensitivity and recognize structural inequality between males and females would be a barrier to developing constructive support for survivors (Leung, 2011). So, it is necessary to provide specific training and education on DV to the service providers to ensure the safe and intensive care of this vulnerable population. Moreover, community programmes are also necessary to increase knowledge and raise awareness about DV against women (Sabri et al., 2015). Based on the findings of this current review, supportive family members and the community were helpful for the survivors in seeking help against violence (Sabri et al., 2018; Shah, 2015; Shamoon, 2018). So, violence prevention and intervention programmes need to engage with family members to help remove barriers and encourage women to seek help. However, there was one drawback present for some immigrant women who faced a number of difficulties due to isolation from the family and the community (Ahmad-Stout, 2021; Mehrotra, 1999; Pallatino, 2018). Community programmes should provide outreach services to effectively help immigrant women who live far from their home country without parents, family members, relatives, friends, or their community. This is also recommended to help recognize barriers and facilitators from both survivors’ and service providers’ perspectives to help government and non-government organizations address DV.

**Table 4.** Implications for Policy, Practice and Research.

**Practice**
Service providers should be trained according to the South Asian context to understand the survivors’ experience with abuse and how better to support them in a culturally sensitive and relevant manner.
The service providers must have expertise in cultural sensitivity and gender-sensitivity assessments and interventions with South Asian ethnic minority women experiencing DV in high-income countries.
Violence prevention and intervention programmes need to arrange for involving the family members, in-laws, husband for breaking the stigma about DV.
Community programmes and education could be the prevention strategies to address cultural norms and Values (which emerged as overarching themes in this review) that may promote and sustain DV in the South Asian community.
**Policy**
Policy should be made for developing culturally appropriate and accessible services in each sector for all South Asian women in all high-income countries which should be compatible with their culture and tradition through multilingual and multiracial services.
Given the importance of religious leaders within the South Asian Community (e.g., minister/imam in islam religion) government organizations and services could work collaboratively with religious leaders to raise awareness and promote education about DV.
The policy should be made to educate statutory and voluntary organizations for understanding the South Asian cultural norms and complexities of the help-seeking behaviors and facilitators of the South Asian women who experienced DV.
The policy should be made to recognize the South Asian cultural norms for statutory and voluntary organizations to ensure survivors confidentiality, achieve their trust, comfort, safety, and security.
**Research**
The future studies should be conducted on the themes in this review, exploring factors such as sociocultural norms, stigma, honor, shame, blame, immigration status, language and financial barriers, which are the unique features/challenges of South Asian communities, and how they influence help-seeking behaviors.
Encourage to explore more comprehensive research areas to develop culturally appropriate interventions to encourage effective help-seeking amongst South Asian women affected by DV.
Need to conduct more research on social support and acculturation of South Asian women who experienced DV and help-seeking barriers in high income countries.

DV = domestic violence.

Policy should be designed to help develop culturally appropriate and accessible services for South Asian women in high-income countries which are compatible with their culture and tradition through multilingual and multiracial services. The policy also needs to be implemented with a proper understanding of South Asian cultural norms interconnected with their religion, ethnic identity, gender identity for both statutory and voluntary organizations, to educate and train all service providers through a multisectoral approach (government DV support staff including police, healthcare staff, social workers, family lawyers, and DV specialists from non-governmental organizations) to ensure survivors’ confidentiality, achieve their trust, comfort, safety, and security. The service providers must have expertise in cultural sensitivity and gender-sensitivity assessments and interventions with South Asian ethnic minority women experiencing DV in high-income countries. Future studies should be conducted on the themes in this review, exploring how factors such as socio-cultural norms, stigma, honor, shame, blame, immigration status, language, and financial barriers influence help-seeking behaviors. The findings can also explore the research areas to develop interventions to encourage effective help-seeking amongst South Asian women affected by DV. It is also needed to conduct more research on social support and acculturation of South Asian women living as immigrants in high-income countries who have experienced DV and help-seeking barriers because of their lack of knowledge and support about host countries. This meta-ethnography identify the need for government and non-government support including family, friends, community, and social support for South Asian women. The findings of this meta-ethnography identify an important knowledge gap that should be addressed to improve the help-seeking behaviors in South Asian women living in high- income countries who have experienced DV.
